# Barriers and Facilitators to Receiving the COVID-19 Vaccination and Development of Theoretically-Informed Implementation Strategies for the Public: Qualitative Study in Hong Kong

**DOI:** 10.3390/vaccines10050764

**Published:** 2022-05-12

**Authors:** Charlene Hl Wong, Claire Cw Zhong, Vincent Ch Chung, Per Nilsen, Eliza Ly Wong, Eng-kiong Yeoh

**Affiliations:** 1 Faculty of Medicine, Jockey Club School of Public Health and Primary Care, The Chinese University of Hong Kong, Shatin 999077, Hong Kong; charlene.wong@link.cuhk.edu.hk (C.H.W.); chenwenzhong@link.cuhk.edu.hk (C.C.Z.); 2 Centre for Health Systems and Policy Research, Jockey Club School of Public Health and Primary Care, The Chinese University of Hong Kong, Shatin 999077, Hong Kong; lywong@cuhk.edu.hk (E.L.W.); yeoh_ek@cuhk.edu.hk (E.-k.Y.); 3 School of Chinese Medicine, The Chinese University of Hong Kong, Shatin 999077, Hong Kong; 4 Department of Health, Medicine and Caring Sciences, Linköping University, SE-581 83 Linköping, Sweden; per.nilsen@liu.se

**Keywords:** vaccine hesitancy, implementation science, COVID-19, qualitative research, patient acceptance of health care

## Abstract

Objectives: enhancing uptake of COVID-19 vaccines is an important tool for managing the pandemic. However, in Hong Kong, the COVID-19 vaccination rate in the general population was unsatisfactory during the early phase of the vaccination program. This two-part study aimed to (i) identify barriers and facilitators to receiving vaccinations, and (ii) develop theoretically-informed implementation strategies for promoting uptake. Methods: in part 1, 45 Hong Kong residents who differed in their willingness to vaccinate (willing (*n* = 15), were unwilling (*n* = 15), and were hesitant (*n* = 15)), were interviewed individually in February 2021. They were invited to express their perceptions of receiving the COVID-19 vaccination. The theoretical domains framework (TDF) was applied to guide the interviews and analyses. Behavioral diagnoses from these findings were then used to develop theoretically-informed implementation strategies in part 2, composed of behavior change techniques (BCTs) informed by the established BCT taxonomy. Results: in part 1, the five main barriers were (i) concerns on severe and long-term side effects; (ii) low confidence in the safety and effectiveness due to concerns of their accelerated development; (iii) unclear information on logistical arrangements of the vaccination program; (iv) insufficient data on safety and effectiveness; and (v) perceived low protection ability conferred by the vaccines. The five main facilitators included (i) healthcare professionals’ recommendations; (ii) news from TV, radio, and newspapers as main sources of trustworthy information; (iii) vaccine-related health education delivered by healthcare professionals; (iv) expectations of resuming to a normal social life; and (v) perceived benefits outweighing risks of mild and short-term side effects. Conclusions: seven implementation strategies were developed in part 2 based on the results above, namely (i) providing trustworthy vaccine-related information and scaling up the promotion; (ii) encouraging healthcare professionals to recommend vaccinations; (iii) giving incentives; (iv) using social influence approaches; (v) allowing a selection of COVID-19 vaccine brands; (vi) increasing accessibility for vaccinations; and (vii) emphasizing social responsibility.

## 1. Introduction

The coronavirus disease 2019 (COVID-19) pandemic continues to pose substantial health and economic burdens worldwide [1]. As of 22 April, 2022, there were more than 500 million confirmed cases of COVID-19, including 6.2 million deaths around the world [2]. To contain the pandemic and prevent new outbreaks, safe and effective COVID-19 vaccines have been regarded as crucial tools [3,4].

As stated by the World Health Organization, vaccine hesitancy was one of the ten threats to global health in 2019 [5]. Public willingness to receive COVID-19 vaccination may thus influence the uptake of COVID-19 vaccination globally. According to a cross-sectional survey involving 4884 respondents in the UK [6], 79.3% were willing to receive a COVID-19 vaccination, while 13.9% were uncertain, and 6.8% refused to receive a vaccination. Another survey among approximately 3200 respondents in China showed that 83.8% were willing to receive a COVID-19 vaccination [7]. In Hong Kong, where the epidemic has been reasonably controlled, public willingness to receive a COVID-19 vaccination ranged from 34.8% to 44.2% [8,9]. The Hong Kong COVID-19 vaccination program was initiated in February 2021 [10]. Two types of COVID-19 vaccines are available for the local public, namely Fosun/BioNTech Comirnaty vaccine and Sinovac CoronaVac vaccine [10]. However, only 20.7% of Hong Kong’s population has received two vaccine doses at the early phase of the program from February to June 2021 [10].

A number of cross-sectional studies have explored factors related to public willingness to receive COVID-19 vaccinations in different countries. Concerns on safety and effectiveness of COVID-19 vaccines were found to be the main reasons for COVID-19 vaccine hesitation among the public in China [11,12], Japan [13], the USA [14], and the UK [6,15]. Due to their cross-sectional designs, findings from these studies may not garner an in-depth understanding of the factors related to the public uptake of vaccinations. At the time of writing, qualitative studies that explore public attitudes towards COVID-19 vaccinations are limited. A UK focus group study among 29 adults showed that the overall attitudes to COVID-19 vaccination were positive due to trust in science. Moreover, they considered the decision to vaccinate as a social norm and necessity for resuming normal social life [16]. Another focus group study involving 43 adults in India indicated that they had mixed perspectives in terms of knowledge, attitude, perceptions, and concerns regarding COVID-19 vaccines [17]. Further qualitative studies are needed to explore more nuanced insights on public attitudes toward such a vaccination. We set out to conduct a two-step qualitative study on this topic in the context of Hong Kong. The first step of this qualitative study aimed to identify barriers and facilitators to receiving COVID-19 vaccinations among the Hong Kong public, while the second step involved developing theoretically-informed implementation strategies according to results from the first step. Implications from these findings may inform local governments and healthcare professionals on how to promote the uptake of COVID-19 vaccinations in Hong Kong, or in countries with unsatisfactory vaccination rates.

## 2. Methods

This study is composed of two parts: Section 2.1—identification of barriers and facilitators to receiving COVID-19 vaccination; and Section 2.2—development of theoretically-informed implementation strategies to improve the uptake of COVID-19 vaccinations.

### 2.1. Identification of Barriers and Facilitators to Receiving COVID-19 Vaccination

In-depth individual qualitative interviews were conducted with 45 Hong Kong residents from 8 February to 12 February, 2021. When the sample size reached 45, data saturation was reached and no new perspectives were generated. This sample size fulfilled the requirement of data saturation (*n* = 16–24) [18] and surpassed the sample size recommendations of qualitative studies for implementation research (*n* = 13) [19]. Qualitative interviews were conducted in Cantonese with the aim of identifying barriers and facilitators to receiving COVID-19 vaccinations among the Hong Kong public.

#### 2.1.1. Study Sampling

A purposive sampling approach was applied to recruit a heterogeneous sample of Hong Kong people who were (i) willing to receive the COVID-19 vaccination (*n* = 15), (ii) unwilling to receive the vaccination (*n* = 15), and (iii) hesitant about whether to receive the vaccination (*n* = 15), so as to capture diversity of perspectives in this study [20]. A person was regarded as vaccine hesitant if one delayed the decision to receive the COVID-19 vaccination in spite of availability [21]. We sampled participants from the community via an investigator’s lay network (CHLW).

Before the interviews began, participants were informed of the study purpose, confidentiality of their contributions, voluntary nature of their participation, and their right of withdrawal at any time. Each individual interview was performed face-to-face or via telephone by two main interviewers (CHLW and CCWZ). Each participant was given a HKD 100 coupon upon completing the interview. Both CHLW and CCWZ are public health researchers with significant experiences in conducting qualitative interviews and analysis. Written informed consent was obtained from all participants. The study was approved by the Survey and Behavioral Research Ethics Committee, The Chinese University of Hong Kong, Hong Kong (reference no.: SBRE-19-595).

#### 2.1.2. Data Collection

**Figure A1:**
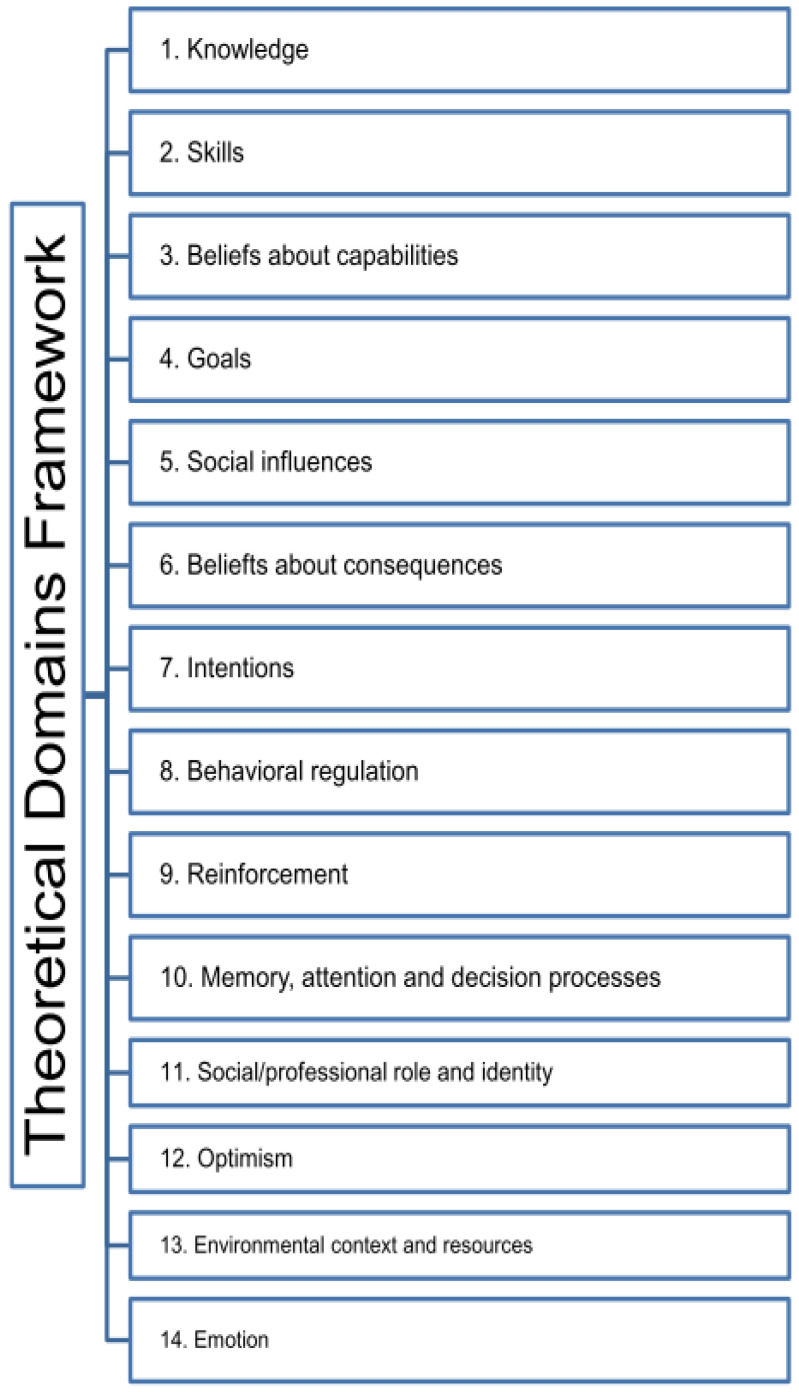
The 14 Domains of TDF.

A semi-structured interview guide (Appendix A) informed by the theoretical domains framework (TDF) [22] was developed by CHLW, CCWZ, and VCHC. TDF is commonly used to identify determinants, including barriers and facilitators, to implement evidence-based interventions (e.g., COVID-19 vaccines) [23]. It is based on a collaboration of behavioral scientists and implementation researchers who identified 33 behavior change theories of relevance for implementation [24]. Constructs from these theories were grouped into 14 domains, thus providing a comprehensive framework of possible barriers and facilitators of behavioral change (Appendix B, Figure A1) [22]. Each interview lasted for 25 to 45 min. During the interviews, CHLW or CCWZ asked the interview questions and took field notes. All interviews were recorded and the content was transcribed verbatim. An audio recording was used to verify interpretations if there were discrepancies in the interview content, with discussions among the investigators.

#### 2.1.3. Data Analysis

The interview transcripts, in the original language of Cantonese, were analyzed in accordance with the 14 TDF domains, iteratively. Two investigators (CHLW and CCWZ) coded the transcribed interviews based on TDF using NVivo software [25]. Important words and sentences in the transcripts were coded into relevant TDF domains through a deductive approach. The words and sentences were summarized into short statements that captured key concepts. The short statements were then used to compare with core concepts within the TDF domains. To minimize researcher bias, the interpretation of statements and coding results were discussed among three investigators (CHLW, CCWZ, and VCHC). Inconsistent coding was amended until consensus was attained [20]. The chosen representative quotes were translated from the interview language of Cantonese to English. In this way, barriers and facilitators to receiving the COVID-19 vaccination among the Hong Kong public within the TDF domains were identified.

### 2.2. Development of Theoretically-Informed Implementation Strategies to Improve Uptake of COVID-19 Vaccination

The identified barriers and facilitators within the TDF domains from Section 2.1 were used to develop theoretically-informed implementation strategies, composed of behavior change techniques (BCTs). The BCT taxonomy is a structured list of 93 techniques that are active elements of interventions targeting to change behavior [26,27]. Mapping of BCTs to the TDF domains has previously been validated [28,29]. Combination of BCTs and TDF has been widely adopted to inform implementation strategy development in different healthcare settings [30,31,32].

Through the intervention-mapping approach, BCTs were first chosen to target determinants within the TDF domains identified in Section 2.1 [28,29]. A comprehensive range of BCTs were then incorporated to generate theoretically-informed implementation strategies [27,28], to improve uptake of COVID-19 vaccination in Hong Kong. The BCT-informed strategies were designed based on the qualitative data collected from study participants in Section 2.1. This ensured the logic and relevance of the strategy design to the vaccine recipients. This intervention-mapping exercise was conducted by CHLW and CCWZ, under the supervision of VCHC, PN, and EKY.

## 3. Results

### 3.1. Identification of Barriers and Facilitators to Receiving COVID-19 Vaccination

#### 3.1.1. Participants

Interviews were conducted with 45 Hong Kong residents who differed in their willingness to receive the COVID-19 vaccination: willing, unwilling, or hesitant about receiving the vaccination. The response rate to invitations was 100%. Of these 45 participants, 28 were females and 17 had chronic disease. Forty-three of them completed secondary education or above. The age distribution of different groups was similar, with 15 participants aged from 18 to 39, 15 aged from 40 to 56, and the remaining 15 aged 60 or above. Detailed sociodemographic characteristics of participants are presented in Table 1.

#### 3.1.2. Barriers and Facilitators

Analysis of the interview data yielded findings that could be mapped into 12 of the 14 TDF domains, except for the domains of skills and behavioral regulation. The content from these 12 domains acted as barriers and facilitators to receiving the COVID-19 vaccination among study participants (numbered 001-045) (Table 2).

#### 3.1.3. Knowledge

Study participants who were willing to receive the COVID-19 vaccination thought that it was very important to obtain knowledge about COVID-19 vaccines: “*I will have more positive attitudes towards COVID-19 vaccination if I know more details about the effectiveness, quality and side effects of the vaccines*” [Interviewee 001].

Insufficient data on safety and effectiveness of the COVID-19 vaccines was regarded as one of the main barriers to vaccinations among the study participants: “*Due to a lack of clinical data, I don’t know which type of COVID-19 vaccine is effective and safe. Therefore, I can’t decide whether I should receive the vaccination or not at the moment*” [Interviewee 003]. Unclear information on the logistical arrangements of the Hong Kong COVID-19 vaccination program was perceived to hamper the willingness to receive the COVID-19 vaccination: “*Sometimes the government leaders state that there will be enough stockpiles of different types of COVID-19 vaccines, but we don’t know when they will exactly arrive in Hong Kong. The timeline of vaccine availability remains uncertain*” [Interviewee 042].

#### 3.1.4. Beliefs about Capabilities

Study participants perceived that vulnerable populations, such as elderly and chronic disease patients, had to bear high risk of adverse events if they decided to receive the COVID-19 vaccination. A chronic disease patient who was unwilling to be vaccinated stated: “*I am a middle-aged woman with hepatitis B, rheumatoid arthritis and high blood pressure. To a chronic disease patient like me, adhering to the precautionary measures is more important to protect myself from COVID-19. I can predict that receiving vaccination would bring me severe side effects*” [Interviewee 028]. Those who perceived themselves as having a low risk of contracting COVID-19, such as young participants, were also less willing to receive the COVID-19 vaccination: “*I am still young, around 30 years old*. *I believe that my immune system would be much better than those aged 60 years or above. My risk of contracting COVID-19 is not that high at all*” [Interviewee 019].

#### 3.1.5. Goals

Some study participants considered receiving the COVID-19 vaccination in order to help reach the societal goal of herd immunity, provided that the vaccines were safe. Participants who were unwilling to receive the COVID-19 vaccination did not believe that herd immunity would be effective enough to protect the public: “*Only some healthcare professionals said that a 70% vaccination rate might lead us to herd immunity. No one knows whether it is true or not. The efficacy rate of various types of vaccines are different, so I don’t think that a 70% vaccination rate can ensure the herd immunity*” [Interviewee 030].

#### 3.1.6. Social Influences

From the study participants’ viewpoints, healthcare professionals played a very significant role in convincing the Hong Kong public to receive the COVID-19 vaccination. Most participants agreed that healthcare professionals’ recommendations on vaccinations, as well as delivery of COVID-19 vaccine-related health education, were main facilitators to the vaccination. Participants expressed that they would be more willing to receive the vaccination if healthcare professionals served as role models (i.e., for receiving vaccinations).

Furthermore, sharing of experiences (on receiving the vaccination) among family members and friends helped promote the vaccination uptake: “*My friends in mainland China have already received COVID-19 vaccination. They didn’t experience any side effects, so I am confident with the safety of COVID-19 vaccines*” [Interviewee 007]. Local government recommendations also increased some participants’ confidence in receiving the vaccination: “*I totally trust our government. The COVID-19 vaccines provided by our government must be safe and effective, so I will receive the vaccines immediately once they are available*” [Interviewee 005].

#### 3.1.7. Beliefs about Consequences

Many of the study participants expressed concerns on severe and long-term side effects of COVID-19 vaccines, such as numbness, chest discomfort, Bell’s palsy, stroke, and even death: “*I am afraid that the COVID-19 vaccines will lead to some unanticipated adverse events like organ failure. Under such circumstance, I can’t work and take care of my family. Therefore, I am not willing to receive the vaccination at the moment*” [Interviewee 021]. Some of them perceived that current COVID-19 vaccines only conferred low protection against COVID-19: “*The virus causing COVID-19 is constantly mutating and becomes more diverse. Some research studies showed that the effectiveness of COVID-19 vaccines against variants was low, so I don’t think that receiving the vaccination can provide us with sufficient protective effect against COVID-19*” [Interviewee 020].

Nevertheless, nearly all participants who were willing to receive the COVID-19 vaccination believed that the vaccines would potentially protect them against COVID-19: “*Although the COVID-19 vaccines can’t be 100% effective, I am confident that it will somehow protect us against developing severe complications*” [Interviewee 002].

#### 3.1.8. Intentions

Many study participants perceived that the potential benefits outweighed risks of mild and short-term side effects of COVID-19 vaccines: “*If the side effects are just fever and headache which last for a few hours or a few days only, I am willing to receive effective COVID-19 vaccines*” [Interviewee 031].

#### 3.1.9. Reinforcement

Adopting the incentives of easing of travel restrictions and relaxation of social distancing measures after receiving the COVID-19 vaccination were perceived to be facilitators: “*I love travelling a lot. If other countries are open to vaccinated Hong Kong travelers, I will receive the COVID-19 vaccination as quickly as possible*” [Interviewee 013]. The study participants agreed that free vaccines would especially motivate people who have financial difficulties to receive the vaccination.

#### 3.1.10. Memory, Attention, and Decision Processes

Study participants highlighted that it was important to have the right to select the types of COVID-19 vaccines themselves. The main criteria for choosing COVID-19 vaccines among the participants were the origins or brands, as well as efficacy rate and potential side effects. One participant who was willing to receive the vaccination stated: “*I will choose the vaccines which are made in China. This is because the vaccine trials are conducted among Chinese who share similar characteristics like us. I am more confident with these vaccines*” [Interviewee 014]. A vaccine-hesitant participant claimed: “*I would choose the vaccines that are made in the US as those pharmaceutical companies have more experiences in developing vaccines against certain diseases. They can provide more detailed data on safety and effectiveness of COVID-19 vaccines as support which increase my confidence*” [Interviewee 035].

#### 3.1.11. Social/Professional Role and Identity

A work environment with a higher risk of COVID-19 exposure was perceived to positively influence the study participants’ willingness to receive the COVID-19 vaccination: “*I am a taxi driver. In case one of my passengers is an asymptomatic patient or has very mild COVID-19 infections, I would be highly vulnerable to COVID-19. To protect myself and my passengers, I* *am willing to receive the vaccination*” [Interviewee 009]. Moreover, several participants indicated that organizational commitment to receiving COVID-19 vaccinations would urge them to get vaccinated: “*I am afraid of losing my job. If my company encourages employees to receive COVID-19 vaccination, I will definitely follow the recommendation and get vaccinated as soon as possible*” [Interviewee 026].

#### 3.1.12. Optimism

Vaccine-hesitant study participants and those willing to receive COVID-19 vaccination believed that getting vaccinated would be the key to resuming normal social life: “*This should be the only solution to fight against COVID-19, and this would reduce infection risk in the short term. Then the pandemic can be controlled and we can go back to normal social life as soon as possible*” [Interviewee 042]. However, nearly all vaccine-hesitant participants and those unwilling to receive the COVID-19 vaccination claimed that they had low confidence in the safety and effectiveness of COVID-19 vaccines due to the rapid and seemingly accelerated development process: “*Based on my understanding, it usually takes 7–8, or even 10 years to develop a new vaccine. I think it is impossible to produce an effective and safe vaccine against COVID-19 within a short period of time like 1 year only*” [Interviewee 044].

#### 3.1.13. Environmental Context and Resources

Study participants usually obtained COVID-19 vaccine-related information from TV, radio, and newspapers: “*The news basically summarize all the existing evidence related to the COVID-19 vaccines. Reporting the latest research findings would increase public confidence towards the vaccine. If healthcare professionals with good understanding of the vaccines can give their advice on these media outlets, I will find it more convincing and subsequently I would be more willing to receive the vaccination*” [Interviewee 002]. Moreover, a short travel distance from home was regarded as an important facilitator for receiving the COVID-19 vaccination: “*I prefer receiving vaccination in a location that is close to my home. I am afraid that I will feel sick right after the vaccination. I will need to go back home immediately after vaccination*” [Interviewee 003].

#### 3.1.14. Emotion

The COVID-19 pandemic posed negative impacts on the emotions of some study participants, negatively influencing their willingness to receive the vaccination: “*Restaurants are only allowed to operate up to 2 people per table until 6 p.m. It is hard to dine out with a group of friends. Meanwhile, I worry that large gathering during the pandemic will increase the infection risk*. *I prefer staying at home all day to protect myself, but I am getting sad and depressed*” [Interviewee 009]. Receiving the COVID-19 vaccination would make the participant feel secure and hopeful in resuming to a normal social life.

### 3.2. Development of Theoretically-Informed Implementation Strategies to Improve Uptake of COVID-19 Vaccination

Through the intervention-mapping approach, a number of BCTs were identified to address the identified barriers and facilitators within the TDF domains to receiving the COVID-19 vaccination among the Hong Kong public. The BCTs were incorporated for generating a total of seven preliminary implementation strategies (Table 3).

#### 3.2.1. Providing Reliable COVID-19 Vaccine-Related Information and Scaling up the Promotion of COVID-19 Vaccination

Detailed information related to the Hong Kong COVID-19 vaccination program, as well as up-to-date local and international clinical data on safety and effectiveness of COVID-19 vaccines, might be delivered via TV, radio, newspapers, and governmental websites. The news from TV, radio, and newspapers were regarded as the main preferred sources of obtaining COVID-19 vaccine-related information among the study participants.

When promoting the Hong Kong COVID-19 vaccination program, it is important to highlight the future benefits of receiving the COVID-19 vaccination. Moreover, COVID-19 vaccine-related health education should be delivered via multiple promotion approaches. For instance, distributing posters and leaflets to the public, advertising in public places and mass media might help promote the importance of receiving the COVID-19 vaccination.

#### 3.2.2. Engaging Healthcare Professionals to Recommend Vaccination for Individuals

Healthcare professionals’ person-centered recommendations on COVID-19 vaccinations might be provided for individuals. They might also emphasize the importance of achieving the goal of herd immunity to the individuals by specifying a sufficient proportion of population receiving vaccination.

#### 3.2.3. Giving Rewards

The Hong Kong government might provide free COVID-19 vaccines within a certain time period. If feasible, the government might also use easing of travel restrictions and relaxation of social distancing measures as rewards to facilitate uptake of COVID-19 vaccinations.

#### 3.2.4. Using Social Influence Approaches

Firstly, healthcare professionals and Hong Kong government leaders could serve as role models for receiving COVID-19 vaccinations. Secondly, people might share experiences on receiving COVID-19 vaccinations with their family members and friends. Lastly, people who have received vaccinations could encourage members of their peer and social groups to join the vaccination program. Psychosocial support groups could be established to reduce negative emotions during the COVID-19 pandemic.

#### 3.2.5. Allowing Selection of COVID-19 Vaccines According to Individual’s Choice

The Hong Kong public might be given the right to select the COVID-19 vaccines. For example, they might choose vaccines of different origins or brands according to their own wills.

#### 3.2.6. Increasing Accessibility of the COVID-19 Vaccination

The Hong Kong government might consider the following factors to increase public accessibility of the COVID-19 vaccination: (i) making vaccination locations more easily accessible; (ii) avoiding crowds and flexible opening hours; and (iii) ensuring availability of different vaccine types in different local districts.

#### 3.2.7. Emphasizing on Social Responsibility

The Hong Kong government might explain to the public that receiving COVID-19 vaccinations is a social responsibility. The government could consider adding COVID-19 vaccinations as a precautionary measure in the workplace, particularly where the environment poses a high risk for transmission, such as taxis and other public conveyances.

## 4. Discussion

### 4.1. Summary of Findings

In part 1 of the study, we identified barriers and facilitators to receiving COVID-19 vaccinations among the Hong Kong public via TDF-guided qualitative interviews. The five main barriers and facilitators are shown in Table 4. Through the intervention-mapping approach in part 2, seven implementation strategies were generated to increase Hong Kong’s public uptake of the COVID-19 vaccination.

### 4.2. Comparison with Current Literature

Some barriers identified among the Hong Kong public in our study are consistent with the determinants associated with COVID-19 vaccine hesitancy in other countries. Concerns on severity and the longer side effects of COVID-19 vaccines are well-established barriers to vaccinations globally [6,11,12,13,14,15,33,34,35]. The public’s lack of confidence in the vaccine’s protective effects against COVID-19, and insufficient knowledge about the safety and effectiveness of vaccines, also lower their willingness to receive COVID-19 vaccinations [34,35]. These barriers may be exacerbated by an expedited vaccine development process and an inadequate level of COVID-19 vaccine literacy among the public [36,37]. Some people believed that the COVID-19 vaccines might not have been properly tested and were rushed into circulation [36,37]. It is noteworthy that the public show preference to COVID-19 vaccines that incur less side effects, rather than high effectiveness [35].

### 4.3. Implications

To facilitate uptake of COVID-19 vaccinations in Hong Kong, the government may consider implementing theoretically-informed strategies as developed in our study. However, such implementation requires support from local governments and healthcare professionals. Details of the three major strategies that address most of the facilitators and barriers, namely (i) providing reliable COVID-19 vaccine-related information and scaling up the promotion of COVID-19 vaccinations; (ii) engaging healthcare professionals to recommend vaccinations for individuals; and (iii) using incentives and social influence approaches, are elaborated as follows:

### 4.4. Providing Reliable COVID-19 Vaccine-Related Information and Scaling up the Promotion of COVID-19 Vaccinations

Firstly, providing reliable COVID-19 vaccine-related information in a clear, transparent, and timely manner can foster public trust in the COVID-19 vaccines [35]. Experiences in Israel, Canada, and the US indicated that transparent monitoring and reporting of adverse events of COVID-19 vaccines were critical in strengthening public confidence toward COVID-19 vaccinations [38,39,40]. Existing studies have highlighted the need for governments to inform the public about both the benefits and harms of receiving COVID-19 vaccinations using data from vaccine trials [33,35,41,42]. This would also help combat misinformation disseminated on social media [33,35,41,42]. Indeed, as a higher proportion of the population is vaccinated, the subsequent fall in COVID-19 incidents may help to dispel anti-vaccination sentiments. A German study indicated that the reporting of numbers, i.e., of people who were vaccinated or expressed a willingness to receive the vaccine, could effectively reduce hesitancy, despite the fact that the initial number of people willing to accept the vaccination was low [43].

### 4.5. Engaging Healthcare Professionals to Recommend Vaccinations for Individuals

Current studies support the idea that healthcare professionals and government leaders’ active participation would facilitate the uptake of COVID-19 vaccinations [38,42]. They play a significant role in scaling up the promotion of COVID-19 vaccinations in Hong Kong. As the perception of accelerated development of vaccines is a major public concern, they should highlight the rigorous standards of the COVID-19 vaccine development process [41] in promotional campaigns in Hong Kong. Moreover, our findings show that young people tend to believe that the potential side effects of receiving COVID-19 vaccinations are greater than the risks of contracting COVID-19. Young people are indeed more vulnerable to spreading COVID-19, given the increased likelihood of socialization and reduced compliance to the precautionary measures against COVID-19 [44,45,46]. Hence, specific promotional campaigns might particularly target young Hong Kong people aged between 25 and 44, with a low vaccine acceptance rate [9]. They are highly encouraged to receive the vaccination in order to maximize the benefits of indirect immunity for the elderly and chronic disease patients [47]. The existing data on safety and effectiveness of COVID-19 vaccines, as well as the severity of COVID-19 among young people, might be emphasized in the campaigns [44].

In addition, healthcare professionals have to provide person-centered recommendations on COVID-19 vaccinations. They should keep their COVID-19 vaccine knowledge up-to-date by obtaining regular information released from the local government [39,48]. However, healthcare professionals may be overloaded with constantly evolving information [48]. As recommended in previous studies, researchers should become involved in health professional teams to distill the latest research findings [39,48]. As knowledge brokers, they might then provide essential information on safety and effectiveness of COVID-19 vaccines for healthcare professionals in a concise manner [39,48]. Meanwhile, healthcare professionals might receive training to improve their communication skills with the public [39,40]. It is expected that collaborations among government, health professionals, and researchers would reduce public confusion [39] and increase public confidence towards COVID-19 vaccinations. It is already known that greater accountability and transparency among politicians and healthcare elites are keys to increase public trust on anti-COVID-19 strategies [49], including vaccine acceptance [43].

### 4.6. Incentive and Social Influence Approaches

Lastly, our study showed that the belief of resuming to a normal social life (by getting fully vaccinated) was one of the main facilitators. To meet public expectations, US and UK experts recommended a return to normal life once a sufficient COVID-19 vaccination rate was achieved [40,41]. Hence, we propose that the Hong Kong government ease travel restrictions and relax social distancing measures as rewards for people who receive the COVID-19 vaccination. As for other rewards, according to the findings of a recent Hong Kong study, the majority of interviewees agreed that a free COVID-19 vaccination should be provided for each permanent resident [50]. Yet, additional financial rewards were not suggested in our data. Substantial research showed that offering cash incentives might make some people view vaccinations as undesirable, with a hidden agenda, or even a dangerous action [40]. People who worry about side effects of COVID-19 vaccines would thus be less willing to receive the vaccination [40].

Social influence by family, friends, and peers may also be keys to encourage vaccine uptake. By emphasizing benefits to others via the creation of herd immunity, which is a public good, COVID-19 vaccination may be branded as a moral obligation [51]. This is because vaccinations would prevent harm to others as well as oneself; the first reason is usually considered as a valid reason for sacrificing personal freedom (i.e., the freedom to choose to not vaccinate). Promoting this rationale via social networks, through support of media campaigns, may be useful, as long as the message is accompanied with reassurance on vaccine safety. The strategy may be particularly useful for targeting population segments in which their workplace environments are associated with higher risks of transmission (e.g., taxi drivers).

### 4.7. Strengths and Limitations

There are some strengths in our study. The use of TDF facilitated identification of barriers and facilitators to receiving the COVID-19 vaccination. Our findings demonstrated the generation of theoretically-informed implementation strategies using the intervention-mapping approach. We achieved a 100% response rate to invitations. This might be because participants were told that their contributions would be useful to inform local policy development regarding the prevention against COVID-19. Hence, they might be more eager to participate in this study. To minimize the perception of coercion, the research team’s professional background was made explicit to the participants before obtaining informed consent [52]. The participants were also informed that their participation was voluntary and they had right to withdraw at any time [53].

However, this qualitative study has several limitations and one of them is a relatively small sample size, although it has satisfied standards in qualitative research. Due to the urgency in informing vaccination promotion strategies, a total of 45 interviewees were recruited from the community. To understand diverse perspectives of the local population, a purposive sampling approach was applied to recruit a heterogeneous sample of Hong Kong residents who differed in their willingness to receive COVID-19 vaccinations: willing (*n* = 15), unwilling (*n* = 15), or hesitant about whether or not to receive the vaccination (*n* = 15). Age distribution of the three groups was also similar, with 15 participants aged from 18 to 39, 15 aged from 40 to 56, and the remaining 15 aged 60 or above. When the sample size reached 45 in this study, data saturation was attained and no new perspectives were generated. It is believed that the study findings from these 45 interviews would offer important insight for the government and healthcare professionals on how to promote the uptake of COVID-19 vaccination in Hong Kong. Moreover, this study was only conducted in Cantonese, which is the most commonly used language by the Hong Kong population [54]. The impact of bias introduced by only interviewing participants in Cantonese would be small, but such findings might not be generalizable to a small proportion of residents who do not speak Cantonese. In addition, the Hong Kong government has been actively promoting the COVID-19 vaccination program. The participants’ views on receiving the COVID-19 vaccination may be distorted by a social desirability bias [55]. To reduce the bias during data collection, the research team assured the participants that their contributions to this study were confidential [55].

## 5. Conclusions

This qualitative study investigated the Hong Kong public’s perception of receiving the COVID-19 vaccination. The five main barriers were (i) concerns of severe and long-term side effects of COVID-19 vaccines; (ii) low confidence in the safety and effectiveness of COVID-19 vaccines due to concerns of the accelerated development and, hence, the benefits of receiving the vaccination; (iii) unclear information on the logistical arrangements of the Hong Kong COVID-19 vaccination program; (iv) insufficient data on the safety and effectiveness of the COVID-19 vaccines; and (v) perceived low protection ability against COVID-19 conferred by the vaccines. The five main facilitators included (i) healthcare professionals’ recommendations on the COVID-19 vaccination; (ii) news from TV, radio, and newspapers as main sources for obtaining COVID-19 vaccine-related information; (iii) COVID-19 vaccine-related health education delivered by healthcare professionals; (iv) belief of resuming to a normal social life by getting fully vaccinated; and (v) perceived benefits outweighing risks of mild and short-term side effects of COVID-19 vaccines. Implications from these findings informed tailoring of implementation strategies via the intervention-mapping approach. The seven implementation strategies included (i) providing reliable COVID-19 vaccine-related information and scaling up the promotion of COVID-19 vaccinations; (ii) engaging healthcare professionals to recommend vaccinations for individuals; (iii) giving rewards; (iv) using social influence approaches; (v) allowing a selection of COVID-19 vaccines according to the individual’s will; (vi) increasing accessibility to COVID-19 vaccinations; and (vii) emphasizing social responsibility. Support from local governments and recommendations for vaccinations from healthcare professionals would be needed to aid the implementation.

## Figures and Tables

**Table 1:** Sociodemographic characteristics of participants (*n* = 45).

Sociodemographic Characteristics	Willing (*n* = 15)	Unwilling (*n* = 15)	Uncertain (*n* = 15)	Total (*n* = 45)
Gender				
- Male	7	4	6	17
- Female	8	11	9	28
Age (years)				
- 18–39	5	5	5	15
- 40–59	5	5	5	15
- ≥60	5	5	5	15
Chronic disease or not				
- With chronic disease	7	5	5	17
- Without chronic disease	8	10	10	28
Education				
- Primary or below	0	0	2	2
- Secondary	9	7	5	21
- Tertiary or above	6	8	8	22
Family monthly household income (HKD)				
- ≤15,000	1	2	0	3
- 15,001–30,000	4	1	4	9
- 30,001–45,000	4	4	2	10
- ≥45,001	6	8	9	23
Living area				
- Hong Kong Island	1	0	1	2
- Kowloon	6	6	4	16
- New Territories	8	9	10	27
Marital status				
- Unmarried	4	4	2	10
- Married/cohabit	10	10	11	31
- Divorce/widow/separated	1	1	2	4
Perceived health status				
- Very good	1	5	2	8
- Good	8	4	8	20
- Fair	6	6	5	17

Keys: willing, participants who are willing to receive COVID-19 vaccination; unwilling, participants who are unwilling to receive COVID-19 vaccination; uncertain, participants who are uncertain about whether to receive COVID-19 vaccination or not.

**Table 2:** Summary of facilitators of and barriers to the COVID-19 vaccination presented under the TDF (*n* = 45).

TDF Domain	Facilitators	Barriers
Knowledge	Knowledge about COVID-19 (*n* = 19)	Unclear information related to the Hong Kong COVID-19 vaccination program (*n* = 23) *
	Professional advice on COVID-19 vaccination are needed (*n* = 18)	Insufficient data on safety and effectiveness of the COVID-19 vaccines (*n* = 19) *
	Knowledge about COVID-19 vaccines (*n* = 18) ^	Knowledge about COVID-19 (*n* = 9) ^&^
	Up-to-date local and international clinical data on safety and effectiveness of COVID-19 vaccines should be provided (*n* = 18) ^@^	
	Sufficient information related to the Hong Kong COVID-19 vaccination program are needed (*n* = 13)	
Beliefs about capabilities	Perceived risk of getting COVID-19 among vulnerable population ^1^ (*n* = 21)	Perceived high risk of adverse events after receiving the COVID-19 vaccination among vulnerable population ^1^ (*n* = 12) ^&^
	Perceived possible risk of contracting COVID-19 (*n* = 14)	Perceived low risk of contracting COVID-19 (*n* = 9) ^ ^&^
	Perceived vulnerability of contracting COVID-19 among healthcare professionals (*n* = 7)	
Goals	Herd immunity against COVID-19 would be considered an incentive (*n* = 9)	Herd immunity against COVID-19 may not be effective enough to protect the public (*n* = 3)
Social influences	Healthcare professionals’ recommendations on COVID-19 vaccination (*n* = 34) *	Low level of trust in the government (*n* = 2)
	COVID-19 vaccine-related health education delivered by healthcare professionals (*n* = 33) *	Family members and friends’ suggestions on the COVID-19 vaccination (*n* = 1)
	Healthcare professionals serve as role models for receiving the COVID-19 vaccination (*n* = 26)	
	Family members and friends’ suggestions/their sharing of experiences on receiving the COVID-19 vaccination (*n* = 17)	
	Government leaders serve as role models for receiving the COVID-19 vaccination (*n* = 14)	
	Government’s suggestions on the COVID-19 vaccination (*n* = 7)	
Social influences (cont.)	COVID-19 vaccine-related health education delivered by government (*n* = 2)	
B eliefs about consequences	Perceived potential in protecting against COVID-19 (*n* = 16) ^	Concerns on severe and long-term side effects of COVID-19 vaccines, such as numbness, chest discomfort, Bell’s palsy, stroke, and even death (*n* = 29) *
	Beliefs of protecting elderly and chronic disease patients against COVID-19 (*n* = 7)	Perceived low protection ability against COVID-19 conferred by the vaccines (*n* = 16) *
	Perceived positive expectations on the effectiveness and side effects of COVID-19 vaccines (*n* = 6)	Concerns on negative impact to family members after receiving the COVID-19 vaccination (*n* = 3)
		Concerns on negative impacts to work after receiving the COVID-19 vaccination (*n* = 1)
Intentions	Perceived benefits outweigh risks of mild and short-term side effects of COVID-19 vaccines (*n* = 30) *	
Reinforcement	Easing of travel restrictions and relaxation of social distancing measures as incentives (*n* = 14)	Easing of travel restrictions and relaxation of social distancing measures as incentives would make public feel negative towards receiving the COVID-19 vaccination (*n* = 1)
	Free COVID-19 vaccines as incentives, particularly for people who have financial difficulties (*n* = 10)	Free COVID-19 vaccines pose concerns on hidden agenda related to promoting vaccination (*n* = 1)
	Cash incentives (*n* = 1)	Cash incentives would further reduce public’s confidence towards COVID-19 vaccines, as it is perceived as a mean to advance a hidden agenda (*n* = 1)
	Purchase insurance for people who are willing to receive the COVID-19 vaccination as an incentive (*n* = 1)	
	Health Care voucher as incentives (*n* = 1)	
Memory, attention and decision processes	Have the right to select types of COVID-19 vaccines according to personal wills (*n* = 27) ^& @^	Fear of needles and allergic reaction to COVID-19 vaccines (*n* = 3)
	Criteria for choosing COVID-19 vaccines: - Origins or brands (*n* = 24) ^@^ - Efficacy rate and potential side effects (*n* = 24) ^@^ - Availability of data related to COVID-19 vaccines concerned (*n* = 18) ^&^ - Consistency of advice provided by healthcare professionals (*n* = 3) - Dosage of COVID-19 vaccines (*n* = 2) - Stockpile of vaccine doses (=1) - Recognition by other countries (*n* = 1)	Previous negative experience of receiving vaccination in chronic disease patients (*n* = 3)
Social/professional role and identity	Work environment with higher risk of COVID-19 exposure (*n* = 11)	
	Organizational commitment to promoting COVID-19 vaccinations (*n* = 7)	
	Leadership/influence on others (*n* = 3)	
	Social responsibility for receiving COVID-19 vaccinations (*n* = 2)	
Optimism	Hope in resuming normal social life by after full vaccination (*n* = 31) *	Low confidence in the safety and effectiveness of COVID-19 vaccines due to concerns on their accelerated development. This has reduced the expected benefits of receiving the vaccination (*n* = 27) *
	High confidence in the benefits of receiving COVID-19 vaccination (*n* = 15) ^	Perceived ineffectiveness in COVID-19 pandemic control despite vaccine availability (*n* = 9) ^@^
	Perceived effective control of the COVID-19 pandemic with mass vaccination (*n* = 14)	Perceived low importance of COVID-19 vaccination (*n* = 2)
		Perceived difficulties in the implementation of the COVID-19 vaccination program (*n* = 1)
Environmental context and resources	Sources of obtaining COVID-19 vaccine-related information: - News from TV, radio, and newspaper (*n* = 34) * - Governmental website (*n* = 6) - YouTube (*n* = 2) - Facebook (*n* = 1)	Low trustworthiness of COVID-19 vaccine-related information (*n* = 6)
	Criteria for determining locations for receiving COVID-19 vaccination: - Short travel distance from home (*n* = 25) - Avoid crowds (*n* = 15) - Opening hours (*n* = 8) - Trust on the organization (*n* = 7) - Availability of vaccine types provided (*n* = 2)	
	High trustworthiness of COVID-19 vaccine-related information (*n* = 17)	
	Negative impact of COVID-19 pandemic (*n* = 10)	
	Online booking to avoid crowds (*n* = 8)	
	Workplace outreach vaccination program (*n* = 1)	
Emotion	Impact of the unpleasant feelings caused by the COVID-19 pandemic triggers willingness to be vaccinated (*n* = 8)	
	Psychosocial support programs for the public during the COVID-19 pandemic are needed to instill confidence in vaccination (*n* = 2)	

Key: * Top five commonly discussed facilitators and barriers to implementation among all participants. ^ Top five commonly discussed facilitators and barriers to implementation among participants who are willing to receive the COVID-19 vaccination. ^&^ Top five commonly discussed facilitators and barriers to implementation among participants who are unwilling to receive the COVID-19 vaccination. ^@^ Top five commonly discussed facilitators and barriers to implementation among participants who are hesitant about receiving the COVID-19 vaccination. ^1^ Vulnerable population, including elderly and chronic disease patients, etc.

**Table 3:** Implementation strategies to maximize the public’s willingness to receive the COVID-19 vaccination.

Implementation Strategies	Details	Relevant BCTs
(1) Providing trustworthy COVID-19 vaccine-related information and scaling up the promotion of COVID-19 vaccination	Channels: • News on TV, radio, newspaper, and governmental website	- Credible source
	• Detailed information related to the Hong Kong COVID-19 vaccination program • Procedures and logistics of vaccination arrangement • Origins or brands and data transparency of COVID-19 vaccines provided • Priority population groups for receiving vaccination • Precautions for those preparing to receive COVID-19 vaccinations	- Action planning
	• Up-to-date local and international clinical data related to COVID-19 vaccines for the public, especially the vulnerable population ✓ Effectiveness in preventing COVID-19 ✓ Incidence of severe adverse events ✓ Monitoring of local vaccination rate	- Information about health consequences - Social comparison - Pros and cons - Vicarious consequences - Review behavior and outcome goals - Information about social and environmental consequences
	• Promotion of the Hong Kong COVID-19 vaccination program ✓ Highlight the future benefits of receiving COVID-19 vaccination, e.g., herd immunity, rapid resumption to normal social life, etc. • Deliver COVID-19 vaccine-related health education ✓ Importance of receiving COVID-19 vaccination: knowledge about COVID-19, how COVID-19 vaccines can control the pandemic and protect vulnerable population, etc. ✓ Potential side effects of receiving the COVID-19 vaccination in short-term and long-term	- Commitment - Vicarious consequences - Information about health consequences - Pros and cons - Comparative imagining of future outcomes - Imaginary reward - Salience of consequences
	• Multiple promotion approaches of the COVID-19 vaccination: ✓ Distributing posters and leaflets to the public ✓ Advertising in public places and mass media (e.g., newspapers, TV, radio, etc.)	- Prompts/cues
(2) Encouraging healthcare professionals to recommend vaccination for individuals	• Healthcare professionals’ recommendations on the COVID-19 vaccination ✓ Person-centered advice for different vulnerable population (e.g., advice stratified by age, occupations, health conditions, etc.) ✓ Emphasize the importance of achieving herd immunity by specifying a sufficient proportion of population receiving vaccination	- Credible source - Pros and cons - Information about others’ approval - Goal setting (behavior and outcome) - Information about social and environmental consequences
(3) Giving rewards	• Providing free COVID-19 vaccines within a certain time period • Easing of travel restrictions • Relaxation of social distancing measures • Purchasing insurance for people receiving COVID-19 vaccination • Distributing health care vouchers • Getting people to return to work faster and revive the economy	- Future punishment - Remove reward - Material incentive and reward (behavior) - Remove punishment
(4) Using social influence approaches	Healthcare professionals and Hong Kong government leaders • Serve as role models for receiving the COVID-19 vaccination Family members and friends: • Sharing experiences on receiving the COVID-19 vaccination	- Information about emotional consequences - Social comparison - Vicarious consequences
	Social support: • People who receive the COVID-19 vaccination may serve as leaders to encourage others to join the vaccination program • Invite people who are willing to receive the COVID-19 vaccination to affirm or reaffirm a strong commitment (using “I will…” statements). • Establishment of psychosocial support groups for the public during the COVID-19 pandemic	- Commitment - Social support (unspecified and emotional) - Information about others’ approval - Reduce negative emotions
(5) Allowing a selection of COVID-19 vaccines according to the individual’s will	Selection criteria include: • Origins or brands • Efficacy rate and potential side effects • Availability of data related to COVID-19 vaccines • Consistency of advice provided by healthcare professionals • Recognition by other countries • Dosage of COVID-19 vaccines • Stockpile of vaccine doses	- Credible source - Pros and cons - Information about health consequences
(6) Increasing accessibility for receiving COVID-19 vaccination	Criteria include: • Making vaccination locations more easily accessible, e.g., workplace outreach vaccination program • Avoiding crowds and flexible opening hours, e.g., online vaccination booking system • Ensuring availability of different vaccine types in different local districts	- Problem solving - Restructuring the physical environment
(7) Emphasizing on social responsibility	• Informing the public that receiving COVID-19 vaccinations is a social responsibility • Adding COVID-19 vaccinations as a precautionary measure under “Health Advice on Prevention of Coronavirus disease (COVID-19) in Workplace” (as stated by the Centre of Health Protection)	- Identity associated with changed behavior - Restructuring the physical environment

Key: BCTs, behavior change techniques. √: Examples of the detailed strategies.

**Table 4:** Top five commonly discussed facilitators and barriers to implementation among all participants (*n* = 45).

Facilitators to Implementation	Barriers to Implementation
Healthcare professionals’ recommendations on the COVID-19 vaccination (*n* = 34)	Concerns on severe and long-term side effects caused by COVID-19 vaccines (*n* = 29)
News from TV, radio, and newspapers as main sources for obtaining COVID-19 vaccine-related information (*n* = 34)	Low confidence in the safety and effectiveness of COVID-19 vaccines due to concerns of their accelerated development, leading to lower expected benefits of receiving the vaccination (*n* = 27)
COVID-19 vaccine-related health education delivered by healthcare professionals (*n* = 33)	Unclear information on logistical arrangements of the Hong Kong COVID-19 vaccination program (*n* = 23)
Expectation of resuming to a normal social life by getting fully vaccinated (*n* = 31)	Insufficient data on safety and effectiveness of the COVID-19 vaccines (*n* = 19)
Perceived benefits outweigh the risks of mild and short-term side effects of COVID-19 vaccines (*n* = 30)	Perceived low protection ability against COVID-19 conferred by the vaccines (*n* = 16)

## Data Availability

The data that support the findings of this study are available upon request from the corresponding author. The data are not publicly available due to privacy or ethical restrictions.

## Appendix A. Semi-Structured Interview Guide Developed Based on the Theoretical Domains Framework (TDF)

1. Do you think that it is important to receive the COVID-19 vaccination? Why?

2. Do you think that it is safe to receive the COVID-19 vaccination? Why?

3. Factors that may influence willingness to receive the COVID-19 vaccination:

*3.1 Domain 1 Knowledge*

(1) How much do you know about COVID-19 (probes: is it a serious, highly contagious disease)? Does your COVD-19 knowledge affect your willingness to receive the COVID-19 vaccination? How?

(2) How much do you know about the COVID-19 vaccines (e.g., vaccine efficacy, vaccine quality, side effects)? Does your COVID-19 vaccine knowledge affect your willingness to receive COVID-19 vaccination? How?

(3) How much do you know about the Hong Kong COVID-19 vaccination program? Is it important to know more details about the program? Do you think that knowing more details about the program would affect your willingness to receive the COVID-19 vaccination? Why?

*3.2 Domain 2 Optimism*

(1) Are you confident in the COVID-19 vaccines? Do you think that the COVID-19 pandemic can be controlled if most of the people received a vaccination? Do these thoughts affect your willingness to receive the COVID-19 vaccination? How?

*3.3 Domain 3 Beliefs about Consequences*

(1) Do you think that the COVID-19 vaccines can protect you and other people? Do these thoughts affect your willingness to receive the COVID-19 vaccination? How?

*3.4 Domain 4 Reinforcement*

(1) Does the provision of free COVID-19 vaccines affect your willingness to receive the COVID-19 vaccination? How?

*3.5 Domain 5 Intentions*

(1) Once the Hong Kong COVID-19 vaccination program is officially launched, would you like to receive the vaccination? Why?

(2) You are informed that receiving the COVID-19 vaccination can effectively prevent COVID-19, but it may bring some side effects. Do the potential side effects affect your willingness to receive the COVID-19 vaccination? What type of side effects would you consider as acceptable? Why?

*3.6 Domain 6 Goals*

(1) Some healthcare experts suggested that herd immunity would be achieved if 70% of the Hong Kong public received the COVID-19 vaccination. Does this goal affect your willingness to receive the COVID-19 vaccination? How?

*3.7 Domain 7 Beliefs about Capabilities*

(1) Are you worried about contracting COVID-19? Which types of people are vulnerable to contracting COVID-19 (e.g., elderly, chronic disease patients)? Do these thoughts affect your willingness to receive the COVID-19 vaccination? How?

(2) Are there any other factors that may affect your willingness to receive the COVID-19 vaccination (e.g., needle phobia, negative experience of getting vaccinated, etc.)? If yes, please elaborate.

*3.8 Domain 8 Memory, Attention and Decision Processes*

(1) What is your previous experience of receiving a vaccination (e.g., flu vaccine/tetanus vaccine/chickenpox vaccine, or other vaccines)? Would your previous experience affect your willingness to receive the COVID-19 vaccination? Why?

(2) Would you like to have the right to select the types of COVID-19 vaccines? Why? If yes, which factors would you consider when selecting the vaccine types? How do these thoughts affect your willingness to get vaccinated?

*3.9 Domain 9 Emotion*

(1) Does the outbreak of COVID-19 in Hong Kong affect your emotions? How (e.g., feeling scared, nervous, or depressed)? Would these emotions affect your willingness to receive the COVID-19 vaccination? If yes, please elaborate.

(2) If there are some psychosocial support programs that can help manage your emotions, would they affect your willingness to receive the COVID-19 vaccination? Why?

*3.10 Domain 10 Environmental Context and Resources*

(1) Does the outbreak of COVID-19 in Hong Kong affect your willingness to receive the COVID-19 vaccination? How?

(2) If you are going to receive the COVID-19 vaccination, where would you prefer to get vaccinated (e.g., community vaccination centers, specific clinics under the Hospital Authority and the Department of Health, private clinics)? Which factors would you consider when determining the locations of getting vaccinated (e.g., travel time, opening hours, etc.)?

(3) Which sources do you rely on to obtain information regarding the COVID-19 vaccination? Are you confident in the sources of information? How do these information sources affect your willingness to receive the COVID-19 vaccination?

*3.11 Domain 11 Social/Professional Role and Identity*

(1) What is the nature of your job? Does the working environment affect your willingness to receive the COVID-19 vaccination? How?

(2) Does your company recommend that you receive the COVID-19 vaccination? If yes, will you follow their recommendations? Why?

(3) If you can influence other people (e.g., family members/friends/colleagues)’s decisions of receiving the COVID-19 vaccination, are you willing to receive the vaccination? Why?

*3.12 Domain 12 Social Influences*

(1) When you are facing a decision about whether to receive the COVID-19 vaccination, with whom would you like to consult? Why?

(2) Whose opinions may influence your willingness to receive the COVID-19 vaccination? Why?

(3) You may discuss with your family members/friends/colleagues/supervisors regarding the COVID-19 vaccination or they may give you some recommendations on this issue, will these affect your willingness to receive the COVID-19 vaccination? Why?

(4) If the government/physicians/nurses/other healthcare professionals provide you some recommendations on the COVID-19 vaccination, will these affect your willingness to receive the COVID-19 vaccination? Why?
